# Optimization of Freeze Drying Conditions for Purified Pectinase from Mango (*Mangifera indica* cv. Chokanan) Peel

**DOI:** 10.3390/ijms13032939

**Published:** 2012-03-06

**Authors:** Amid Mehrnoush, Shuhaimi Mustafa, Abdul Manap Mohd Yazid

**Affiliations:** 1 Department of Food Technology, Faculty of Food Science and Technology, Universiti Putra Malaysia, 43400 UPM Serdang, Selangor, Malaysia; E-Mail: Mehrnoush_amid@yahoo.com; 2 Department of Microbiology, Faculty of Biotechnology and Biomolecular Science, Universiti Putra Malaysia, 43400 UPM Serdang, Selangor, Malaysia; E-Mail: Shuhaimi@biotech.upm.edu.my

**Keywords:** freeze drying, mango peel, pectinase, response surface methodology, yield

## Abstract

Response surface methodology (RSM) along with central composite design (CCD) was applied to optimize the freeze drying conditions for purified pectinase from mango (*Mangifera indica* cv. Chokanan) peel. The effect of pectinase content (−2.66, 62.66 mg/mL), Arabic gum (−1.21, 10.21%, w/v), and maltodextrin (0.73, 7.26%, w/v) as independent variables on activity, yield, and storage stability of freeze-dried enzyme was evaluated. Storage stability of pectinase was investigated after one week at 4 °C and yield percentage of the enzyme after encapsulation was also determined. The independent variables had the most significant (*p* < 0.05) effect on pectinase activity and yield of the enzyme. It was observed that the interaction effect of Arabic gum and maltodextrin improved the enzymatic properties of freeze-dried pectinase. The optimal conditions for freeze-dried pectinase from mango peel were obtained using 30 mg/mL of pectinase content, 4.5 (%, w/v) of Arabic gum, and 4 (%, w/v) of maltodextrin. Under these conditions, the maximum activity (11.12 U/mL), yield (86.4%) and storage stability (84.2%) of encapsulated pectinase were achieved.

## 1. Introduction

In terms of production and consumer acceptance, mango is universally a very important and popular tropical fruit and tops the super fruit list [1]. Mango belongs to the *Mangifera* genus, a family of flowering fruit-bearing trees known as Anacardiaceae, which also includes several varieties of other tropical fruit trees [2]. The major by-product of processing mango into some products such as nectar, juices, pickles, and canned slices, is mango peel. Mango peel is rich in enzymes such as pectinase, amylase, xylanase, protease, peroxidase and polyphenol oxidase [3]. Thus, mango peel can be used as a valuable, economic and abundant media source for the commercial production of natural enzymes [4]. Pectinase is one of the upcoming fruit enzymes which break down pectin bounds in plant tissue [5]. There are some reports on the application of pectinase in the textile industry for fiber crops degumming, and for the production of good quality paper. In addition, fermentation of tea and coffee, extraction of oil and clarification of fruit juice are other applications of the enzyme in the food industry. Enzymes are unstable and follow many inactivation pathways [6] thus; some of them are formulated by encapsulation method to avoid inactivation during storage [7]. There are several methods for the encapsulation of this product in the food industry such as spray drying, spray chilling, coacervation, fluidized bed coating, extrusion and liposome entrapment [8]. Because enzymes are very sensitive and easily denatured, freeze-drying is one of the best methods for preservation of biomolecules and should be employed for their encapsulation [9,10]. However, during freeze drying some proteins in these enzymes could be denatured due to the crystallization of the solvent water that loosens the hydrophobic bonds of protein resulting from broken intramolecular hydrogen bounds and distribution of the protein shell [11]. Thus, some stabilizers should be employed to decrease deactivation and destabilization of freeze-dried enzyme [12]. Maltodextrin is now widely used in the food industry in the form of a bodying agent, coating, and carrier [13] because it has good solubility, low viscosity at high solid content, and provides oxidative stability to encapsulated enzyme. Therefore, maltodextrin as a good coating agent was employed for pectinase encapsulation. Also, gum Arabic, with its solubility, low viscosity, emulsification qualities and excellent retention of volatile compounds, is very versatile for purposes of encapsulation. Its wall material makes it ideal for the encapsulation of enzyme because it is both a surface active agent and drying matrix [14]. Therefore, this study investigates the effect of two coating agents, namely, Arabic gum and maltodextrin on enzymatic properties of pectinase. Since there is not any literature about optimization of freeze-drying conditions of pectinase from mango peel, response surface methodology (RSM) was employed in this study to model the possible relationship between the enzyme encapsulation variables and enzymatic properties of encapsulated pectinase.

## 2. Results and Discussions

### 2.1. Response Surface Analysis

Table 1 shows the estimated regression coefficients of the response surface models and the corresponding *R^2^* and adjusted *R*^2^ values. As presented in the table, the significant (*p* < 0.05) response surface models with high *R*^2^ and adjusted *R*^2^ value varied from 0.944–0.997 and 0.972–0.989, respectively and were obtained for all response variables studied. In addition, no significant (*p* > 0.05) lack of fit was observed for the equations, and it can therefore be deduced that the models of response surface were suitably and accurately used for predicting high variation percentage (≥80%) of the properties of the pectinase in relation to the encapsulation variables. Table 2 also indicates the linear, quadratic and interaction effects of pectinase content (X_1_), Arabic gum (X_2_) and maltodextrin (X_3_) on each of the response variables. The significance of the *p*-value and F-ratio determined are also shown in Table 2. Results (Table 2) show that the independent variables had the most significant (*p* < 0.05) effect on activity, yield and storage stability of encapsulated pectinase from mango peel.

### 2.2. Activity of Pectinase

**Figure 1:**
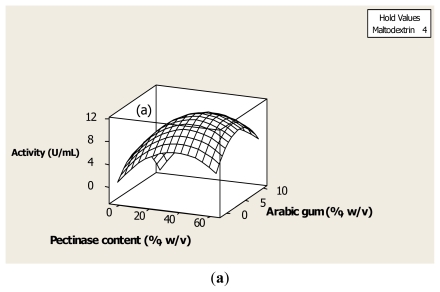

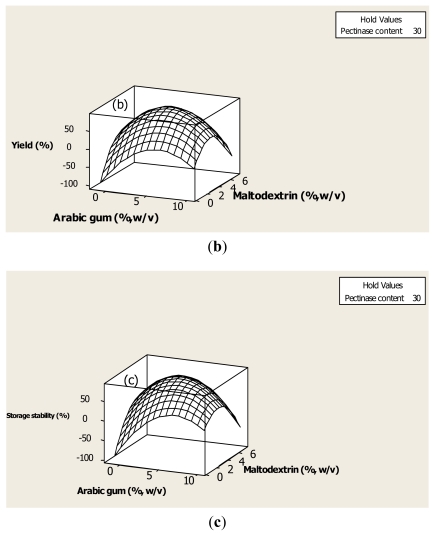
Response surface plots showing the significant (*p* < 0.05) interaction effects of encapsulation factors on enzymatic properties of pectinase. (**a**) Surface plot of pectinase activity *versus* Arabic gum, pectinase content (U/mL); (**b**) Surface plot of yield (%) *versus* maltodextrin and Arabic gum; (**c**) Surface plot of storage stability (%) *versus* maltodextrin and Arabic gum.

The pectinase activity was significantly (*p* < 0.05) influenced by the main effect of pectinase content, maltodextrin, Arabic gum and the quadratic effects of all independent variables as well as the interaction effect of pectinase content and Arabic gum and interaction effect of Arabic gum and maltodextrin (Table 2). The interaction effect of pectinase content and Arabic gum showed the most significant (*p* < 0.05) effect on pectinase activity. It should be considered that the wall to core ratio is one of the most important parameters for retention of core during encapsulation and it could affect the formation and release characteristics of encapsulated enzyme. As shown in Figure 1a, the pectinase showed the highest enzyme activity in 30 mg/mL of pectinase content while the enzyme activity was decreased at higher or lower amounts than this. Coating agents also indicated the positive effect on activity of the enzyme. Arabic gum made the enzyme more rigid and decreased the hydrophobia of enzyme and increased the water mobility in freeze-dried product. Maltodextrin also showed good solubilityin sodium hydrogen phosphate at pH 4.3, low viscosity (5.07 ± 0.03 mPas) at high solid content and provides oxidative stability to encapsulated enzyme. A similar observation was reported by Mireille Alloue *et al.* [15] who were working on encapsulation of lipase from *Yarrowia lipolytica*. The highest amount of pectinase activity (Y2 = 11.12 U/mL) was predicted to be at a combined level of 30 mg/mL of pectinase content, 4.5% (w/v) of Arabic gum and 4% (w/v) of maltodextrin during freeze-drying of the pectinase.

### 2.3. Yield of Pectinase

The results (Table 2) indicated that the main effect of pectinase content, Arabic gum and maltodextrin as well as the interaction effect of Arabic gum and maltodextrin had the most significant (*p* < 0.05) effect on yield of pectinase. Yield of enzyme is obtained based on the ratio of pectinase after encapsulation to that before encapsulation (Equation (1)). Therefore, an increase in pectinase activity causes an increase in the yield of the enzyme. Encapsulation of pectinase in Arabic gum provides an example of swelling-control for the control release of enzyme. It means that Arabic gum swells to the absorption of buffer, thus pectinase in the swollen part is diffused out of the Arabic gum [16]. Arabic gum for controlled release of the enzyme enhances the activity of pectinase. It was seen that Arabic gum and maltodextrin caused increased yield of pectinase in order to increase the enzyme activity. As shown in the results (Table 2), the interaction effect of Arabic gum and maltodextrin showed that the most significant (*p* < 0.05) effect on yield of enzyme because the mixture of Arabic gum and maltodextrin indicated high solid content and acceptable pectinase activity. Maltodextrin also as an effective coating agent confirms the protective effect against protein deneturation [17]. Izutsu *et al.* [18] have also arrived at a similar conclusion in freeze-drying of β-galactosidase using additives. From Figure 1b, it can be seen that the interaction effect of 4.5% (w/v) of Arabic gum and 4% (w/v) of maltodextrin provided the highest yield (86.4%) of freeze-dried pectinase.

### 2.4. Storage Stability of Pectinase

One of the desirable outcomes of freeze drying optimization is the higher stability of the enzyme during storage. The results indicate that stability of pectinase is significantly increased in the presence of coating agents. Based on the results (Table 2) the main effect of Arabic gum and maltodextrin show the most significant (*p* < 0.05) effect on pectinase stability. Storage stability of pectinase is considerably increased whereas Arabic gum improves the stabilizing properties of the enzyme by protective steric barrier around core material [19]. In addition, Arabic gum reactivates the enzyme after storage by increasing enzyme water activity. It should be noted that water activity of enzyme is one of the important factors that keeps enzymes active and stable. The deactivation and destabilisation of enzymes can be achieved by increasing the water activity upon water uptake [20]. Also, 4% (w/v) of maltodexrin increases the stability of enzyme because this coating agent has the ability to form glasses, therefore, enhancing the enzyme stability in order to lower the mobility of core in the wall. This means that the protective effect of maltodextrin preserved the amorphous structure of pectinase during freeze drying. As such, core could be protected through coating agents (wall) during encapsulation. As clearly shown in Figure 1c, the highest value of storage stability was achieved at 5% of maltodextrin. Anandaraman and Reineccius [21] and Wagner and Warthesen [22] also reported that storage stability of core materials was significantly increased after encapsulation when maltodextrin was used. Therefore, it can be concluded that the stability of encapsulated pectinase is about 84.2% when core material, Arabic gum and maltodextrin concentration are in the ratio of 30 mg/mL, 4.5% (w/v) and 4% (w/v), respectively.

### 2.5. Optimization Procedure

The target of optimization is to achieve optimum levels of independent variables that result in desirable response goals so the individual and overall optimization procedures were performed to achieve this target. To determine the optimum region, numerical and graphical optimization procedures were employed. Graphical optimization (using 3D surface plot) was used to determine the optimum region and numerical optimization was employed to determine the exact optimum points. In addition, a comparison was made between the experimental data and predicted value due to investigate the adequacy of the response surface equations [23]. Based on the results, the response variables for pectinase activity, yield and storage stability were predicted to be 11.12 U/mL, 86.4% and 84.2%, respectively.

### 2.6. Validation of Model

**Figure 2:**
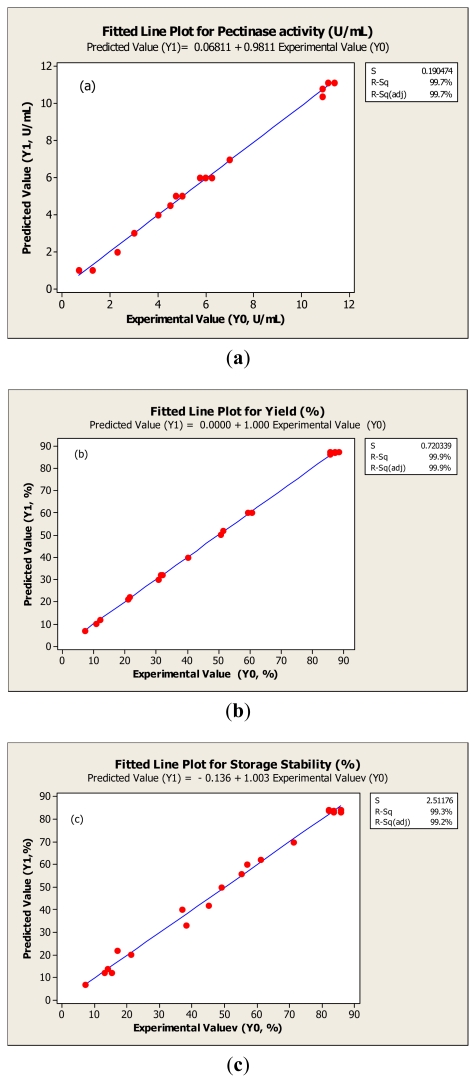
Fitted line plot indicating the closeness between predicted values (Y1) and experimental value (Y0) for pectinase activity (**a**), yield (**b**) and storage stability (**c**).

The adequacy of the response surface equations is indicated by a comparison between the experimental value and the predicted data. The comparison is done by generating a fitted-line plot (with experimental values on X-axis and predicted values on Y-axis) for the results obtained, showing how close it is to or how far it deviates from the fitted line. Figure 2a–c shows high *R*^2^ (>0.95) for fitted line plots and overall closeness of these variables, thus indicating that the response surface model is adequate for predicting the varied enzymatic properties as functions of the conditions in freeze drying.

## 3. Experimental Section

### 3.1. Material

Mango fruits (*Mangifera Indica* cv. Chokanan) used in this study were purchased from a local market in Selangor, Malaysia in slightly under-ripened commercial maturity stage with a Brix value of 14. All chemicals and reagents used were analytical grade. Pectin from citrus fruits, bovine serum albumin (BSA), 3,5-dinitrosalicylic acid (DNS) and Bradford reagent were supplied by Sigma Chemical Co. (St. Louis, MO, USA). Disodium hydrogen phosphate anhydrous, sodium hydrogen phosphate monohydrate and Arabic gum were purchased from Merck (Darmstadt, Germany). Maltodextrin with dextrose equivalence (DE) 10 was obtained from San Soon Seng Food Industries (Selangor, Malaysia).

### 3.2. Preparation of Feedstock

One hundred and five kilograms of mango were washed with distilled water and then were peeled with stainless steel knife. Subsequently, mango peel (five hundred grams) was cut into small pieces (3 mm × 3 mm × 1 mm) and blended with a commercial laboratory blender 32BL79 (Dynamic Corporation of America, Torrington, CT, USA) in 50 mL of sodium hydrogen phosphate (pH 4.3) at 4 °C for 4 min. Following this, the homogenate sample was filtered using cheesecloth to remove plant tissue.

### 3.3. Purification of Crude Extract

Aqueous two-phase system (ATPS) based polyethylene glycol (PEG) and salt was used to purify pectinase from mango peel. The highest purification factor (13.2) and yield (96.7%) of pectinase was achieved in concentration of 14% (w/w) PEG 4000 and 14% (w/w) potassium phosphate at pH 7.0 with addition of 3% (w/v) of NaCl to 25% (w/w) of feedstock [24].

### 3.4. Freeze Drying of Pectinase

Different concentrations of Arabic gum (−1.21, 10.21%, w/v) and maltodextrin (0.73, 7.26%, w/v) were prepared and added to (−2.66, 62.66 mg/mL) of purified pectinase. Subsequently, the mixtures were frozen at −40 °C for 24 h before the freeze drying. Then, frozen samples were lyophilised at −40 °C for 24 h on a VIRTIS Genesis freeze dryer.

### 3.5. Pectinas Activity Assay

The pectinase activity was determined according to the procedure of Miller [25] with some modifications. First of all encapsulated enzyme was dissolved in sodium hydrogen phosphate at pH 4.3 to eliminate of coating agents. The reaction mixture contained 0.5 mL of enzyme solution, 0.5 mL of 50 mM sodium acetate buffer (pH 4.3) and 1 mg/mL pectin. The mixture was incubated in a water bath at 50 °C for 30 min and 3 mL DNS was added to stop the reaction. A spectrophotometer (575 nm) was used in the determination of pectinase activity while the reducing sugar release was measured by galactouronic acid as standard. In addition, the protein contents of samples were determined using dye binding method as described by Bradford [26] and BSA was used as standard.

### 3.6. Yield

Yield of pectinase was determined by dividing pectinase activity after freeze drying (E) to activity of enzyme before encapsulation (F) and multiplying of result to 100 [27].

(1) Yield=(E/F)×100

### 3.7. Storage Stability

The storage of purified pectinase was for a predetermined period of one week at a temperature of 4 °C. Storage efficiency, which is the ratio of enzyme activity after storage to its initial activity multiplied 100, determined the storage stability (Equation (2)) [28].

(2) Storage stability=(Activity of enzyme after encapsulation/Initial enzyme activity)×100

### 3.8. Statistic Design

The response surface methodology (RSM) has been identified as an important method in the statistical design. Collection of mathematical and statistical method useful for experimental design, optimization procedure and analysis of data is RSM [23]. Application of this method in the development, designing and new scientific formulation is very important [29]. RSM is widely used in biological, industrial and physical food science and engineering sciences. This method is employed where several independent variables have most effect on response variables. RSM includes the experimental design for exploring of independent variables, and finding the optimized values of independent variables that create desirable response variables [30]. Thus, RSM using central composite design was used to investigate the effect of three independent variables for encapsulation of pectinase namely pectinase content (−2.66, 62.66 mg/mL), Arabic gum (−1.21, 10.21%, w/v) and maltodextrin (0.73, 7.26%, w/v) on pectinase activity (Y_1_), yield of enzyme (Y_2_) and storage stability (Y_3_) of pectinase from mango peel. It should be noted, the corresponding experimental design and the concentration of coating agents range were considered based on preliminary studies. Twenty purified enzymes were encapsulated based on central composite design, including eight factorial points, six axial points (±α) and six center points (Tables 3 and 4). There was a six-time repetition of the center point to determine the possibility of pure error [31]. The polynomial regression equation was applied to investigate response surface behavior to achieve the response equation (Y). The RSM model is explained below (Equation (3)):

(3) $${\text{Y}}_{0}=\beta_{0}+\beta_{1}{\text{x}}_{1}+\beta_{2}{\text{x}}_{2}+\beta_{3}{\text{x}}_{3}+\beta_{11}{{\text{x}}_{1}}^{2}+\beta_{22}{{\text{x}}_{2}}^{2}+\beta_{33}{{\text{x}}_{3}}^{2}+\beta_{12}{\text{x}}_{1}{\text{x}}_{2}+\beta_{13}{\text{x}}_{1}{\text{x}}_{3}+\beta_{23}{\text{x}}_{2}{\text{x}}_{3}$$

where, Y is the response variable, β_0_, β_1_, β_2_, β_3_, β_11_, β_22_, β_33_, β_12_, β_13_ and β_23_ are the regression coefficient for constant, linear, quadratic and interaction effect, respectively. The adequacy of polynomial equations was investigated by determination of coefficient (*R*^2^) [32]. Meanwhile, *R*^2^ of at least 0.80 is proof of a good model fit while larger values of absolute *t*-value and smaller values of *p*-value show that the variables will be more significant (*p* < 0.05). In addition, in order to determine the adequacy of the response surface equations, a comparison was made between the experimental data and predicted values from the reduced response regression [33].

## 4. Conclusions

In this study, response surface methodology was used to investigate the main and interaction effects of important independent variables on activity and stability of pectinase from mango peel. Main and interaction effect of pectinase content, Arabic gum and maltodextrin showed a significant (*p* < 0.05) effect on activity and yield of the enzyme; thus these independent variables should be kept in all reduced response surface models. This study, also demonstrated that Arabic gum and maltodextrin as coating agents protected pectinase against probable denaturation which is caused by changes in the structure and state of solvent water in the enzyme during freeze drying. Furthermore, core to wall ratio considerably affected the rate of core release from microencapsules and core retention during encapsulation. For this reason, pectinase content should be considered as an important parameter in encapsulation procedure. The results showed that the highest activity (11.12 U/mL), yield (86.4%) and storage stability (84.2%) of pectinase was achieved using 30 mg/mL of pectinase, 4.5% (w/v) of Arabic gum and 4% (w/v) of maltodextrin. The most important finding in this study was that the protection of enzymes can be effectively achieved with the use of coating agents in freeze-drying.

## Figures and Tables

**Table 1:** Regression coefficients *R*^2^, adjusted *R*^2^ and probability values of the response surface models.

Regression Coefficient	Pectinase Activity (Y_1_, U/mL)	Yield (Y_1_, %)	Storage Stability (Y_3_, %)
b_0_	87.13	11.12	83.68
b_1_	8.50	1.75	3.50
b_2_	6.00	0.25	6.00
b_3_	6.44	0.49	6.44
b_1_^2^	5.68	1.68	6.67
b_2_^2^	26.40	1.69	24.99
b_3_^2^	26.88	3.60	20.98
b_12_	6.00	0.37	6.00
b_13_	1.50	1.00	1.50
b_23_	9.00	0.25	14.00
*R*^2^	0.994	0.997	0.991
*R*^2^ (*adj.*)	0.981	0.989	0.972
Regression (*p*-value)	0.000 a	0.000 a	0.000 a
Lack of fit (*p*-value)	0.106 b	0.322 b	0.262 b

b_i_: The estimated regression coefficient for the main linear effects; b_i_^2^: The estimated regression coefficient for quadratic effects; b_ij_: The estimated regression coefficient for the interaction effects; 1: Pectinase content; 2: Arabic gum; 3: Maltodextrin;

a Significant (*p* < 0.05);

b No significant (*p* > 0.05).

**Table 2:** F-ratio and *p*-value for each independent variable effect in the polynomial response surface models.

Variables		Main Effects			Quadratic Effects			Interaction Effects		

		X_1_	X_2_	X_3_	X_1_^2^	X_2_^2^	X_3_^2^	X_1_X_2_	X_1_X_3_	X_2_X_3_
Pectinase	*p*-value	0.001 *	0.000 *	0.011 *	0.003 *	0.004 *	0.003 *	0.002 *	0.372	0.000 *
Activity (Y_1_, U/mL)	F-ratio	30.80	147.86	15.36	29.5	34.56	19.83	274.56	0.963	27.87

Yield (Y_2_, %)	*p*-value	0.000 *	0.001 *	0.000 *	0.033 *	0.019 *	0.002 *	0.178	0.009 *	0.000 *
	F-ratio	528.54	69.88	104.24	10.24	14.28	54.02	2.65	22.84	564.06

Storage Stability (Y_3_, %)	*p*-value	0.004 *	0.000 *	0.000 *	0.200	0.010 *	0.042 *	0.016 *	0.410 *	0.000 *
	F-ratio	24.80	108.16	139.94	2.18	16.72	4.36	12.88	0.79	70.05

X_1_, X_2_ and X_3_: The main effect of pectinase content, Arabic gum and maltodextrin, respectively; X_1_^2^, X_2_^2^ and X_3_^2^: The quadratic effect of pectinase content, Arabic gum and maltodextrin, respectively; X_1_X_2_: The interaction effect of pectinase content and Arabic gum; X_1_X_3_: The interaction effect of pectinase content and maltodextrin; X_2_X_3_: The interaction effect of Arabic gum and maltodextrin.

* Significant (*p* < 0.05).

**Table 3:** Level of independent variables established according to central composite design (CCD).

Independent Variable Levels	Independent Variables Levels				

	Axial (−α)	Low	Center	High	Axial (+α)
Pectinase content (mg/mL)	−2.66	10	30	50	62.66
Arabic gum (%, w/v)	−1.21	1	4.5	8	10.21
Maltodextrin(%, w/v)	0.73	2	4	6	7.26

**Table 4:** Matrix of central composite design (CCD).

Treatment	Block	Pectinase Content (mg/mL)	Arabic Gum (%, w/v)	Maltodextrin (%, w/v)
1^a^	1	30.00	4.50	4.00
2^a^	1	30.00	4.50	4.00
3	1	10.00	8.00	6.00
4	1	10.00	1.00	2.00
5	1	50.00	1.00	6.00
6	1	50.00	8.00	2.00
7	2	30.00	4.50	0.73
8	2	30.00	4.50	7.26
9	2	30.00	10.21	4.00
10	2	62.66	4.50	4.00
11^a^	2	30.00	4.50	4.00
12^a^	2	30.00	4.50	4.00
13	3	30.00	−1.21	4.00
14	3	−2.66	4.50	4.00
15^a^	3	30.00	4.50	4.00
16	3	10.00	1.00	6.00
17	3	10.00	8.00	2.00
18	3	50.00	1.00	2.00
19^a^	3	30.00	4.50	4.00
20	3	50.00	8.00	6.00

a centre point.
